# Development and validation of a predictive model of acute glucose response to exercise in individuals with type 2 diabetes

**DOI:** 10.1186/1758-5996-5-33

**Published:** 2013-07-01

**Authors:** Bryan S Gibson, Sheri R Colberg, Paul Poirier, Denise Maria Martins Vancea, Jason Jones, Robin Marcus

**Affiliations:** 1 Veterans Affairs Medical Center, Salt Lake City, UT, USA; 2 University of Utah, Salt Lake City, UT, USA; 3 Old Dominion University, Norfolk, VA, USA; 4 Institut universitaire de cardiologie et de pneumologie de Québec, Quebec, Canada; 5 University of Pernambuco, Pernambuco, Brazil; 6 Kaiser Foundation Health Plan/Hospital, Pasadena, CA, USA

**Keywords:** Prediction, Exercise, Type 2 Diabetes, Blood Glucose

## Abstract

**Background:**

Our purpose was to develop and test a predictive model of the acute glucose response to exercise in individuals with type 2 diabetes.

**Design and methods:**

Data from three previous exercise studies (56 subjects, 488 exercise sessions) were combined and used as a development dataset. A mixed-effects Least Absolute Shrinkage Selection Operator (LASSO) was used to select predictors among 12 potential predictors. Tests of the relative importance of each predictor were conducted using the Lindemann Merenda and Gold (LMG) algorithm. Model structure was tested using likelihood ratio tests. Model accuracy in the development dataset was assessed by leave-one-out cross-validation.

Prospectively captured data (47 individuals, 436 sessions) was used as a test dataset. Model accuracy was calculated as the percentage of predictions within measurement error. Overall model utility was assessed as the number of subjects with ≤1 model error after the third exercise session. Model accuracy across individuals was assessed graphically. In a post-hoc analysis, a mixed-effects logistic regression tested the association of individuals’ attributes with model error.

**Results:**

Minutes since eating, a non-linear transformation of minutes since eating, post-prandial state, hemoglobin A1c, sulfonylurea status, age, and exercise session number were identified as novel predictors. Minutes since eating, its transformations, and hemoglobin A1c combined to account for 19.6% of the variance in glucose response. Sulfonylurea status, age, and exercise session each accounted for <1.0% of the variance. In the development dataset, a model with random slopes for pre-exercise glucose improved fit over a model with random intercepts only (likelihood ratio 34.5, p < 0.001). Cross-validated model accuracy was 83.3%.

In the test dataset, overall accuracy was 80.2%. The model was more accurate in pre-prandial than postprandial exercise (83.6% vs. 74.5% accuracy respectively). 31/47 subjects had ≤1 model error after the third exercise session. Model error varied across individuals and was weakly associated with within-subject variability in pre-exercise glucose (Odds ratio 1.49, 95% Confidence interval 1.23-1.75).

**Conclusions:**

The preliminary development and test of a predictive model of acute glucose response to exercise is presented. Further work to improve this model is discussed.

## Introduction

Exercise has been widely recognized to ameliorate insulin resistance and hyperglycemia in individuals with type 2 diabetes mellitus (T2DM) [1,2]. Studies that have examined the acute effects of moderate exercise on blood glucose in T2DM have generally demonstrated an immediate, lowering effect of exercise on blood glucose levels [3-7].

To date, only one research group has attempted to develop a predictive model of the acute blood glucose response to exercise in individuals with T2DM [8]. Jeng and colleagues examined the relationship of pre-exercise blood glucose, exercise duration, percentage of Age Adjusted Maximum Heart Rate (% AAMHR), and sex on blood glucose changes with treadmill walking [8]. They reported that three of the four predictors (pre-exercise blood glucose, exercise duration, and % AAMHR) were significant. Their predictive model accounted for 37% of the variance in glucose changes with exercise. In a separate study, similar results were found when individuals performed arm exercise; however % AAMHR was not a significant predictor in this case [9].

Our ultimate goal is to develop and validate a practically implementable predictive model of acute glucose response to exercise. More specifically, we plan to develop a model based on information that individuals with T2DM should have available (e.g., latest hemoglobin A1c), or that they could collect with tools at their disposal (e.g., self-monitored blood glucose). Such a model could be useful to individuals by serving educational and motivational purposes, particularly if it were readily available in their daily lives (e.g., embedded in a mobile phone application). This project encompassed preliminary work toward that goal: We used data from prior trials to identify significant predictors of blood glucose change, and test potential mixed-effect model structures. We then tested the resulting model in a second, prospectively captured test dataset.

## Research design and methods

### Data for model development

We aggregated data from three prior diabetes and exercise studies to determine the significant predictors and to test potential model structures. These prior studies were conducted in Virginia, USA [10]; Sao Paolo, Brazil [11]; and Quebec, Canada [12]. The aggregated dataset represents 56 individuals with T2DM performing 488 exercise sessions. All individuals were taking an oral diabetes medication and had complete data records for the following variables: 1) pre-exercise blood glucose (measured no more than five minutes prior to the start of exercise); 2) post-exercise blood glucose (measured within five minutes of exercise termination); 3) age; 4) sex; 5) hemoglobin A1c; 6) metformin status (dummy coded); 7) sulfonylurea status (dummy coded); 8) exercise session number; 9) minutes since last meal; 10) exercise duration; and 11) % of age adjusted maximum heart rate (% AAMHR) during exercise. Additional file 1 includes a table describing the three original datasets that were combined to create the development dataset.

### Data for testing the model

The data used to test the model were collected between December 2009 and November 2011 from participants in a supervised community exercise group at the University of Utah. For this analysis, we included data from participants taking an oral diabetes medication who had complete records on the variables identified as significant in the model development phase. While data on individuals’ baseline physical activity levels was not available, program staff stated that, similar to the trials data, the majority of participants reported in engaging in little to no physical activity in the 12 months preceding enrollment.

Since both datasets were de-identified and the data collected from the diabetes exercise group at the University of Utah was routinely collected on all participants, this study was approved with a waiver of informed consent by the University of Utah Institutional Review Board.

### Assessment of model error in relation to accuracy of glucose measurements

The glucometers used in both the retrospective dataset for model development and the prospective dataset for model testing are designed for individual self-monitoring. These glucometers are required to meet the International Organization for Standardization (ISO) specification for measurement error: ± 0.83 mmol/L if blood glucose is less than 4.2 mmol/L and ± 20% otherwise [13]. Therefore, model error in this study was defined as an error greater than the measurement error of these devices.

### Transformation of minutes since eating

Based on prior evidence suggesting that the glycemic lowering effect of exercise is greater postprandially than pre-prandially [14], a dummy variable was created for the postprandial state (≤ 120 minutes since eating when exercise began vs. >120 minutes).

We also performed a non-linear transformation of minutes since eating. The first component of this transformation was intended to model the variation in postprandial insulin levels [15]. The second component of the transformation was intended to model the increasing effect of counter-regulatory hormones as the time since eating increased beyond 180 minutes. The effect of these hormones is to promote glycogenolysis and therefore a smaller decrease, or even an increase, in glucose compared to postprandial exercise [4,16].

For the transformation, we first divided the minutes since eating variable into two vectors. The first vector was the range of minutes since eating ≤180. This was multiplied by *π*, sine transformed, and normalized from 0–1. The second vector was the range of minutes since eating >180. This vector was normalized from 0 to 1 and multiplied by -1. The two vectors were then recombined (see Additional file 1 for graph displaying the transformed variable in relation to the variable prior to transformation).

### Determination of significant predictors

The following twelve variables were candidate predictors: pre-exercise blood glucose, age, sex, hemoglobin A1c, metformin status (dummy coded), sulfonylurea status (dummy coded) exercise session number, minutes since last meal (as a linear predictor), non-linear minutes since meal, post-prandial state, exercise duration, and percent of age adjusted maximum heart rate during exercise (% AAMHR).

For variable selection, we used a mixed-effects LASSO (Least Absolute Shrinkage Selection Operator) procedure. As with ordinary multivariable linear regression, the LASSO minimizes the sums of squares, but does so contingent upon the sum of the absolute values of the model coefficients being less than a tuning parameter, S. The result of this penalization is that some model coefficients are constrained to zero while the absolute value of other coefficients increase [17]. The mixed-effect LASSO accounts for the repeated measures within subjects, and the unbalanced structure of the data (i.e., varying number of exercise sessions for individual subjects).

Since our goal was to use the LASSO procedure to identify novel predictors, we “forced” the predictors previously identified to be significant by Jeng at al. (pre-exercise glucose, % AAMHR, and exercise duration) (8), into the model by including them unpenalized in the LASSO. We then systematically decreased the penalization constant, in 0.1 decrements beginning from a point at which only the unpenalized predictors were included, to a value of 0 (no penalization). From the set of potential models output by the LASSO, we selected the model for which the Bayesian Information Criterion (BIC) statistic was minimized.

### Determination of relative importance of predictors

Using the predictors that remained with non-zero coefficients after the LASSO, we applied the Lindemann Merenda and Gold (LMG) algorithm to estimate the relative importance of each of the predictors. This algorithm calculates the contributed proportion of variance explained for each predictor averaged over orderings among predictors [18]. Since this algorithm does not account for repeated measures within subjects, only data from the first exercise session was used for this estimation.

### Rationale for mixed effects model

The data used in this analysis encompasses variation at two levels: variation between subjects and variation within subjects (repeated measures of blood glucose levels from the same individual). We chose a mixed effects model because it can account for these two forms of variability and improve the estimation of population level (fixed) effects [19]. In addition the accuracy of these models improves as the individual contributes more data, supporting our goal of developing a practically useful model [20].

### Testing of model structure

We used a series of likelihood ratio tests to compare the baseline mixed model (random intercepts only; grouped by subject ID) to more complex models. These tests were done sequentially, based on the relative importance of predictors estimated by the LMG algorithm. Predictors that vary within subjects were modeled as random slopes (e.g. individual-specific coefficients for pre-exercise glucose). Predictors that vary between individuals were modeled as grouping factors for random intercepts (e.g. individuals grouped within levels of hemoglobin A1c). Within the lme package in R, random effects are estimated as components of the full model using the expectation–maximization algorithm [21].

### Cross validation of model in development dataset

The resulting model was then tested in a leave one out cross-validation using the development dataset. We calculated model error as the percentage of predictions that were within measurement error.

## Testing of the model

To test the predictive accuracy of the model in the test dataset, we iteratively partitioned the test data set into training sets and test sets. For each subject and exercise session, a training set was created, which included data available prior to the exercise session of interest for that particular subject, and all available data for all other subjects. The model was constructed using this training set. The predicted change in glucose was calculated based on the subject and session of interest (test set) and compared to the actual change in glucose. This approach allowed the use of all of the data without creating a biased estimate of predictive performance.

We assessed the model’s accuracy as the percentage of predictions within measurement error. We calculated model accuracy for all predictions and by prandial state. To estimate the proportion of variance explained (R^2) by the model in the test dataset, we used a method recently proposed by Nakagawa and Schielzeth [22]. This method allows for independent estimation of the proportion of variance explained in a mixed–effects model by the fixed effects and also by the full model (combined fixed and random effects). However, it does not allow for estimation of R^2 in models with multiple levels of random effects (e.g. random slopes within random intercepts). Therefore, we used a model with only random intercepts for these calculations.

We assessed the model’s practical utility by the number of individuals with ≤1 model error after the 3rd exercise session. Finally, we assessed model error across individuals graphically.

### Post-hoc assessment of predictors of model error

Since our analysis pointed to significant inter-individual variability in model performance, we conducted a post-hoc analysis to see if we could determine a priori, whom the model might work for. To this end, we created a mixed-effects logistic regression model including variables that were not accounted for in the predictive model. These variables were: sex, metformin status, and the standard deviation of the individuals’ pre-exercise glucose and post-exercise glucose levels in the exercise sessions prior to the test set. The output of this model was the odds of a model error. Odds > 1 indicating a higher likelihood of model error compared to individuals with average values for the predictors in the model. Odds < 1 indicating a decreased likelihood of model error compared to individuals with average values for the predictors in the model.

### Software used

All analyses were performing using R, statistical computing software [23]. For the LASSO variable selection, we used the lmmlasso package [24]. To calculate the relative importance of each predictor variable (i.e., the LMG algorithm), we used the relaimpo package [18]. The mixed effect predictive models were implemented using the lme function in the nlme package [25]. For the mixed effects logistic regression model we used the Zelig package [26].

## Results

Tables 1 and 2 describe the categorical and continuous predictors used in this study. Table 1 presents the categorical data used in the study as counts (percent). Differences between the development and test datasets for categorical variables were tested using Chi-square tests. Table 2 presents the continuous variables as mean (± standard deviation, SD). Differences between the development and test datasets for each continuous variable were tested using Wilcoxon rank-sum tests.

**Table 1:** Categorical descriptors of the individuals and exercise sessions

**Predictor name**	**Data used for model development**	**Data used for model testing**	**Chi-square Test**
	**Counts (percent)**	**Counts (percent)**	**(*P *value)**
Sex	38 Males (67.8)	23 males (48.9)	0.08
Sulfonylurea	39 = Yes (69.6)	18 = Yes (38.3)	<0.01
Metformin	34 = Yes (60.7)	44 = Yes (93.6)	<0.01
Postprandial state	392 postprandial (80.3)	157 postprandial (36.0)	<0.001

**Table 2:** Continuous descriptors of the individuals and exercise sessions

**Predictor name**	**Data used for model development**	**Data used for model testing**	**Wilcoxon Rank-sum test**
	**Mean (±SD)**	**Mean (±SD)**	**(*P *value)**
Age (years)	54.3 (7.9)	55.9 (9.7)	0.58
HbA1c (%)	7.1 (1.8)	6.9 (1.1)	0.86
Pre-exercise glucose (mmol/L)	9.5 (3.3)	7.8 (2.7)	<0.001
Exercise duration (min)	44.7 (14.6)	34.9 (10.3)	<0.001
Percent Age-Adjusted Maximum Heart Rate (% AAMHR)	72.5 (3.7)	75.5 (8.7)	<0.001
Time since meal (min)	121.5 (138.5)	182.3 (99.9)	<0.001

The values of the categorical and continuous variables in Tables 1 and 2 might be considered typical values of individuals with T2DM treated with oral medications, and the ranges indicative of the heterogeneity in this population.

### Predictors selected

Table 3 presents the variables selected by the LASSO procedure using the development dataset. The coefficients of the model for which the Bayesian information criteria were minimized are presented in the second column. The proportion of the variance explained by each predictor and the 95% confidence interval for that estimate (as calculated by the LMG algorithm), are presented in the third column. A large proportion of the variation in glucose changes was explained by pre-exercise glucose levels (30.6%), followed by minutes since eating (modeled three different ways: as a linear predictor, a non linear predictor, and a dichotomous prandial state), which accounted for 16.0% of the variance on average. Hemoglobin A1c explained another 3.6% of the variance on average. The remaining five variables together explained 5.0% of variance on average.

**Table 3:** Parameter estimates from the LASSO and estimated proportion of variance explained

**Variable**	**Coefficient**	Proportion of variance explained*
	**(Development dataset)**	**(Bootstrap derived 95% CI**^**1**^**)**
Pre-exercise glucose*	-0.46	30.6 (13.6-45.2)
Minutes since eating	0.002	7.9 (3.2-17.2)
Non linear minutes since eating (range -1 to 1)	-0.1	4.9 (2.6-10.3)
Hemoglobin A1c	0.24	3.6 (1.7-13.3)
Postprandial status	1.2	3.2 (1.9-8.1)
Exercise duration*	-0.024	1.7 (0.7-9.1)
Percentage of maximum heart rate*	1.83	1.5 (0.3-11.0)
Sulfonylurea	-0.2	1.0 (0.2-6.2)
Age	-0.004	0.8 (0.2-7.9)
Exercise session number	-0.06	NA**

1. *CI* Confidence Interval* These variables were unpenalized in the LASSO **Since the LMG algorithm does not account for repeated measures, only data from the first exercise session was used and therefore the relative contribution of this variable could not be assessed.

### Testing of model structure

Table 4 presents the results of the series of likelihood ratio tests we conducted to test potential model structures. The addition of random slopes for the variable pre-exercise glucose significantly improved the model fit over a random intercepts-only model (Likelihood ratio 34.5, *P* < 0.0001). The additions of other variables, as either random slopes or intercepts, were not significant.

**Table 4:** Tests of potential model structure using Likelihood ratio tests

**Variable**	**Likelihood ratio**	**Significance (*P *value)**
Pre-exercise glucose	34.5	0.0001**
Minutes since eating	4.3	0.23
Non linear minutes since eating (range -1 to 1)	4.2	0.25
Hemoglobin A1c	0.04	0.99
Postprandial status	1.5	0.68
Exercise duration	1.4	0.71
Percentage of maximum heart rate	0.25	0.97
Sulfonylurea	0.12	0.99
Age	0.1	0.99
Exercise session number	3.6	0.31

### Cross validated model error: development dataset

In cross-validation testing, the model predicted a change in glucose with exercise within measurement error for 83.3% of predictions in the development dataset. Model error varied by prandial state: for sessions where the individual was postprandial, the accuracy was 82.4%. Conversely, for exercise sessions during which the individual was pre-prandial, model accuracy was 87.8%.

### Testing the model

#### ***Model structure: test dataset***

The model we tested using the test dataset implemented the predictors and model structure determined using the development dataset but did not use the estimated coefficients from that model. We chose this approach for two reasons. First, our planned implementation of the model will involve refitting the model as individuals contribute more data; therefore we simulated this situation in testing the model. Second, the two datasets differed significantly across multiple predictors (Tables 1 and 2); using the coefficients from the model fit in the development dataset would have unnecessarily compromised model accuracy in the test dataset.

#### ***Predicted vs. actual glucose change***

**Figure 1:**
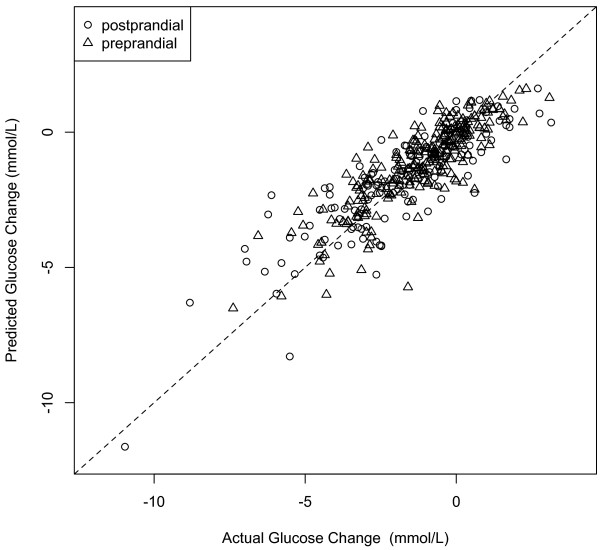
Predicted glucose change vs. actual glucose change for exercise sessions performed in pre-prandial and postprandial states in the test dataset.

Figure 1 presents model predictions vs. actual glucose change in the test dataset. This figure suggests that model errors are distributed relatively uniformly across the range of outcomes. Overall the model predicted exercise related change in glucose levels with 80.2% accuracy. Similar to the results in the development dataset, model accuracy differed based on individual’s prandial state, with pre-prandial (83.6%) predictions being more accurate than postprandial predictions (74.5%).

The proportion of variance explained (R^2) by the model in the test dataset was assessed for the fixed effects alone, and for full the model. The fixed-effects components of the model accounted for 66.8% of the variance in change in glucose levels, while the full model (fixed effects and random intercepts) accounted for 74.7% of the variance.

In our assessment of overall model utility, 31/47 subjects had ≤1 model error after the third exercise session. For the remaining 16 individuals, the number of model errors after the third exercise session ranged between two and five additional errors.

#### ***Model error by subject***

**Figure 2:**
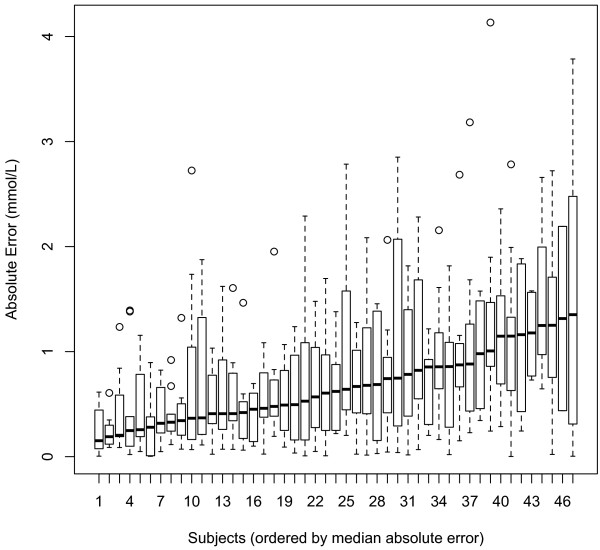
Distributions of subject-specific absolute model error in the test dataset (box represents interquartile range, whiskers represent 1.5 * interquartile range).

Figure 2 presents model error by subject for the test dataset. In this plot, each box represents an individual. Subjects are ordered by their median absolute error so that boxes on the left are those for whom the model performs well and boxes on the right are those for whom the model performs poorly (Figure 2). From this plot it is evident that model errors are not uniformly distributed and the model performs better for some individuals over others.

#### ***Predictors of model error***

To investigate the heterogeneity in model accuracy across subjects evident in Figure 2, we used a mixed effect logistic regression to examine the relationship between individual attributes and model error. In this model, the within-subject standard deviation in pre-exercise glucose levels was the only variable which was associated with model error (Odds ratio =1.49, 95% Confidence Interval = 1.23-1.74, *P* = 0.003). To assess the practical significance of this finding, we recreated the boxplot of model error by individuals and ordered the individuals by their within-subject standard deviation in pre-exercise glucose. This plot is included in the Additional file 1 and suggests that variability in pre-exercise glucose is not practically useful to discriminate individuals for whom the model predicts accurately from those for whom the model is inaccurate.

## Discussion

In this study, we used exercise trials data to identify novel predictors of acute glucose response to exercise, examine the relative importance of selected predictors, and test potential model structures. We then used prospectively obtained data to evaluate the predictive accuracy of the developed model. Finally, we assessed the association between model error and individuals’ attributes.

This work led to interesting results. Apart from the known predictors of acute glucose change with exercise (i.e., pre-exercise glucose, % AAMHR and exercise duration), we identified minutes since eating, hemoglobin A1c, age, sulfonylurea status, and exercise session number as additional novel predictors. Our results regarding the relative importance of these predictors may attest to their practical significance. For example, minutes since eating and hemoglobin A1c together accounted for 19.2% of the variance on average, suggesting that these are important predictors that should be included in future models. Conversely sulfonylurea status, age, and exercise session number each accounted for 1.0% or less of the variance in glucose change, indicating that although they are statistically significant, their practical utility as predictors may be limited. The heterogeneity of model error across individuals led us to an important question: do we know whom the model might work for? We attempted to address this question by assessing the relationship between individual attributes and model error. We found a statistically significant, but practically weak, association between intra-individual pre-exercise glycemic variability and model error. Therefore in this study we could not *a priori* determine whom the model might work for. Potential explanations for this between-subject variability in model accuracy include differences between individuals in exposures (e.g. medication regimens), and genetics [27] that were unaccounted for by this study.

Implementation of a model such as the one developed and tested in this study will require an information system that captures patient generated data (e.g. self monitored glucose), integrates it with clinical data (e.g. HbA1c, medications) and uses the data to generate a personalized prediction. With this in mind, after further development of this model we intend to implement it within a mobile-accessible integrated personal health record. The model output will be presented as a change in a simulated glucose curve [28] and this interactive simulation will be presented in a manner that would both educate and motivate the user [29]. Users will be given the opportunity to "play" with different exercise routines, observe the predicted effects on glycemia, and plan their own activity to meet their goals.

### Strengths

This study had several strengths. First, we used data from a geographically diverse sample of individuals and rigorous statistical methodologies to identify predictor variables, and assess their relative importance. We believe these methods minimize the likelihood that our identified predictors and model structure are anomalous findings. Second, the model was tested in a separate, prospectively collected dataset to establish its predictive accuracy and practical utility. While the model was less accurate than we might have hoped for, these results provide a benchmark for future iterations of the model. Finally, the relationship between model error and individuals’ attributes was investigated to address the question of whom the model might work for. This enabled us to identify limitations of the model and develop research questions for future work.

### Limitations

This study has limitations that we plan to address in future work. Measurement of glucose was performed with a glucometer intended for individuals’ self-monitoring. Our overarching goal was to develop a model that could be used by individuals with diabetes, who rely on self-monitored glucometer values to evaluate changes in glucose levels in their daily lives. Given this contingency, our methods were appropriate, however, it would be preferable to assess model accuracy using a more stringent reference standard. A second limitation was the limited nature of the dataset used for model development. For example, the model did not differentiate resistance vs. aerobic exercise and did not include data on the content of the subjects’ latest meal. We hypothesize that inclusion of these variables may improve the accuracy of future versions of the model. A final problem was that the data used in this study was observational and therefore not balanced across prandial states or exercise intensities. As a result we were not able to fully assess the role of prandial state and exercise intensity as predictors. In future work we plan to address these limitations by experimentally manipulating and balancing predictors within individuals and using a more accurate blood glucose measurement method.

In summary, we developed and validated a model to predict acute exercise induced changes in blood glucose levels in individuals with type 2 diabetes. We are encouraged by these preliminary results and encouraged that with further work a model based on this work might be useful in clinical practice and in individuals’ self-management of their disease. We believe the method and approach used in this study generalize to the area of personalized healthcare interventions.

## Competing interest

None of the authors reported any conflict of interest related to this study.

## Authors’ contributions

BG recruited primary researchers to contribute their data, conducted the statistical analyses and drafted the manuscript; SC contributed data to the development dataset, and drafted the manuscript, PP contributed data to the development dataset, and drafted the manuscript; DMV contributed data to the development dataset, and drafted the manuscript; JJ oversaw the statistical analyses and drafted the manuscript; RM contributed data to the development dataset and the test dataset, and drafted the manuscript; All authors read and approved the final manuscript.

## Supplementary Material

Additional file 1 Includes: 1) a graphic of the non-linear transformation of minutes since eating. 2) descriptions of the three datasets aggregated to create the development dataset. 3) an analysis of model error by participant sorted by intra-individual glycemic variability. Click here for file
