# Supplementary material for: Development and validation of a predictive model of acute glucose response to exercise in individuals with type 2 diabetes
Source: Diabetol Metab Syndr. 2013 Jul 1;5:33. doi: 10.1186/1758-5996-5-33 (PMC3701573; doi:10.1186/1758-5996-5-33)

Online Supplement

Figure 1. Below depicts the nonlinear transformation of the variable minutes since meal in relation to the original variable.

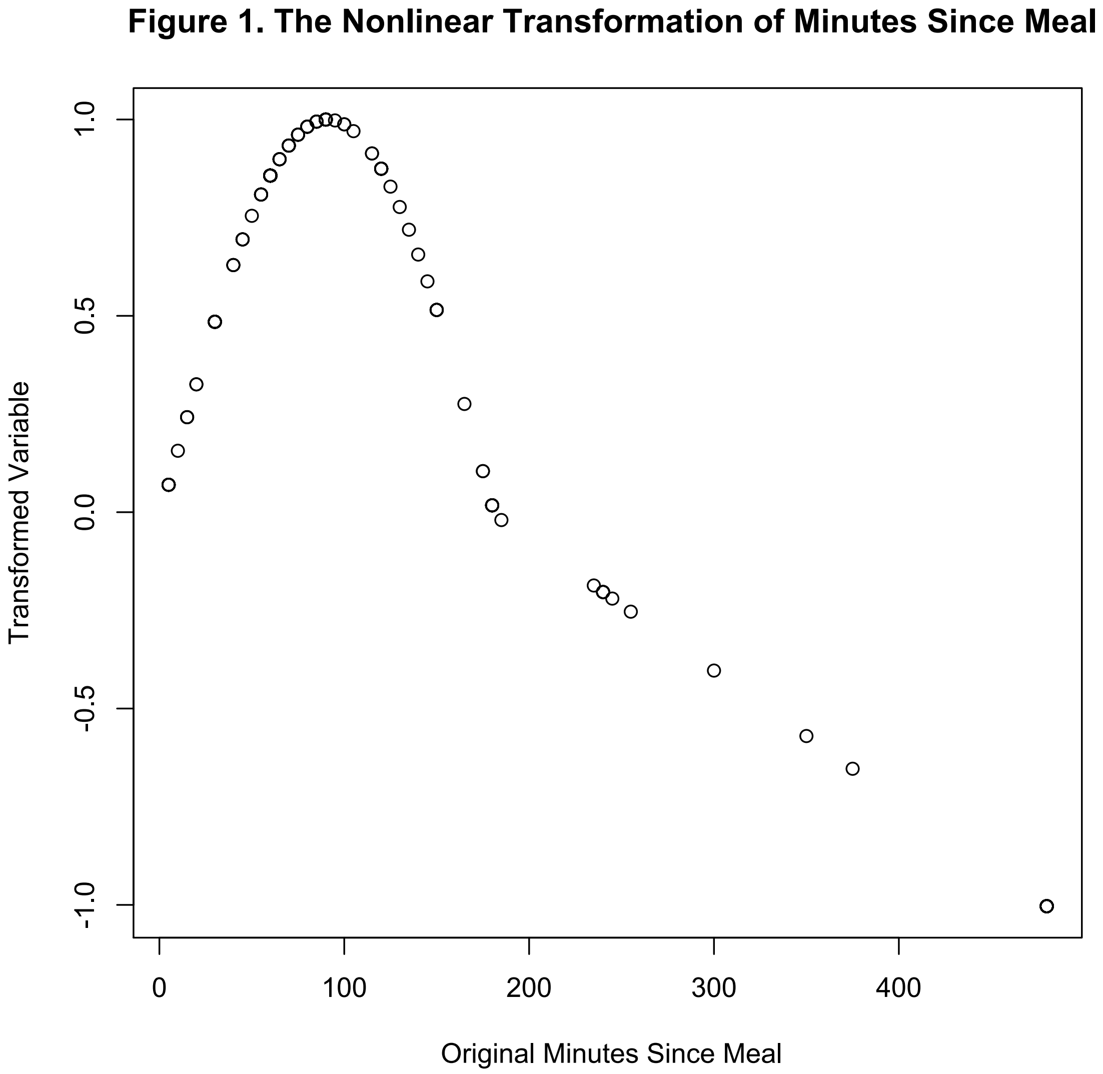

Tables 1 and 2 describe the distributions of the categorical and continuous predictors in the individual datasets that were aggregated to create the development dataset. In Table 1 values are counts (percentage), in table 2 values are means (standard deviation).

Table 1. Distribution of categorical predictors in individual datasets that were aggregated into the development dataset

Variable	Colberg 2009 [10] (9 subjects, 18 sessions)	Vancea 2009 [11] (19 subjects, 190 sessions)	Gaudet-Savard 2007 [4] (28 subjects, 280 sessions)
**Sex**	5 males (55%)	5 males (26.3%)	28 males (100%)
**Sulfonylurea**	5 yes (55%)	11(57.8%)	23 (82.1%)
**Metformin**	6 yes (66.6%)	14 yes (73.6%)	14(50%
**Prandial state at start of exercise session**	9 post prandial (50%)	190 post prandial (100%)	193 post prandial (68.9%)

Table 2. Distribution of continuous predictors in individual datasets that were aggregated into the development dataset

Variable	Colberg 2009 [10] (9 subjects, 18 sessions)	Vancea 2009 [11] (19 subjects, 190 sessions)	Gaudet-Savard 2007 [4] (28 subjects, 280 sessions)
**Age (yrs.)**	59.5 (9.0)	58.5 (5.5)	51.1 (7.7)
**Hemoglobin A1c (%)**	6.8 (1.1)	8.0 (1.9)	6.4 (1.5)
**Exercise duration (min)**	30 (0)	30 (0)	55.5 (9.6)
**Exercise heart rate (% AAMHR)**	70.6 (4.4)	70.0 (0.1)	74.4 (3.9)
**Minutes since meal (min)**	92.5 (90.0)	60.0 (0)	165.5 (168.8)
**Pre-exercise glucose (mmol/L)**	7.6 (2.3)	8.5 (2.9)	10.3 (3.3)

Figure 2. Below depicts the distribution of errors by individual. In this figure individuals are ordered by the mean of the standard deviation of their pre-exercise glucose values. As described in the manuscript, the standard deviation in pre-exercise glucose was calculated in each iteration of the model using data up to, but not including the test session. This figure suggests that variability in pre-exercise glucose is only weakly associated with model error and will not effectively discriminate individuals for whom the model might work and those for whom it will not.

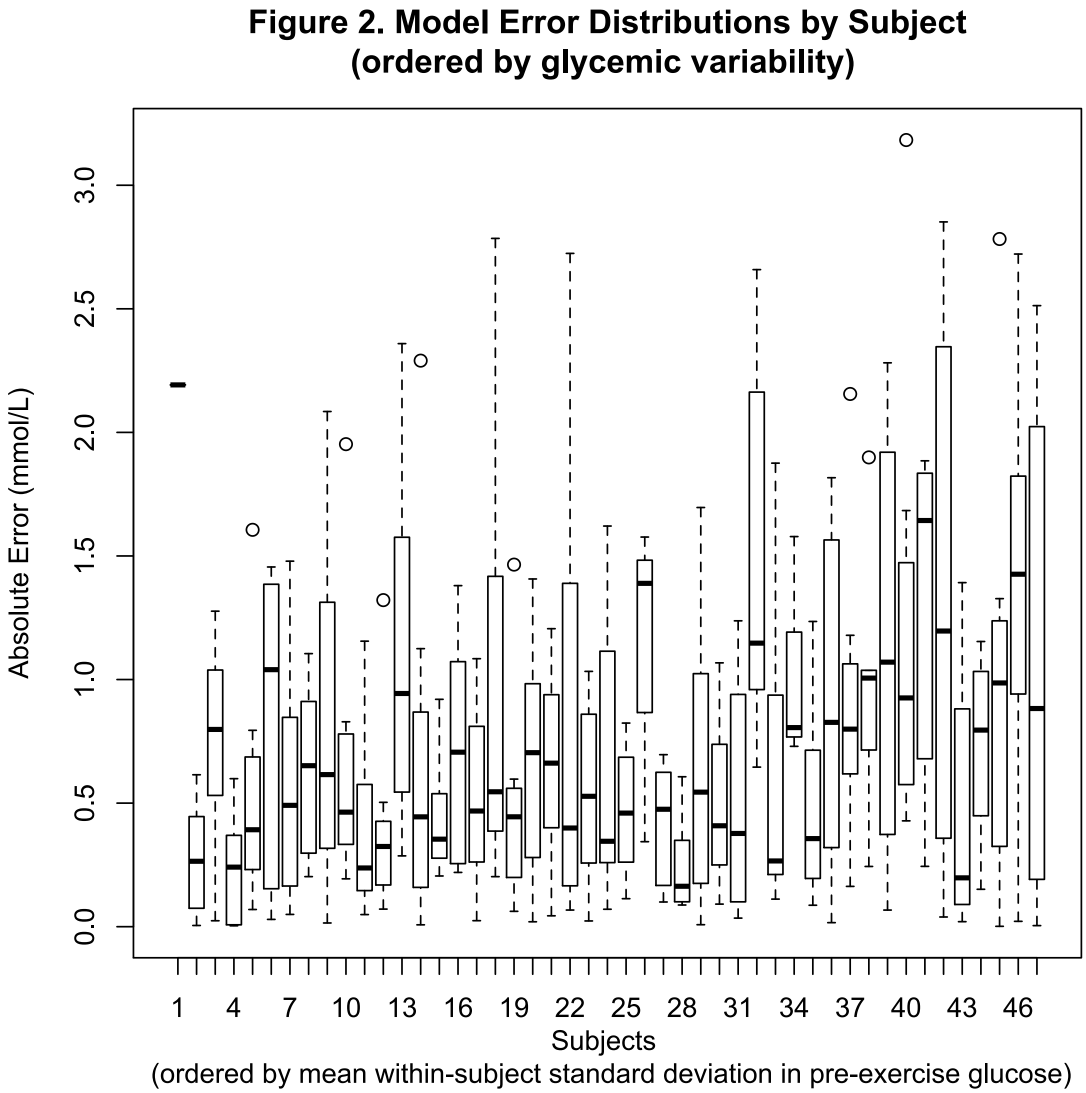

Supplement: Additional file 1 — Includes: 1) a graphic of the non-linear transformation of minutes since eating. 2) descriptions of the three datasets aggregated to create the development dataset. 3) an analysis of model error by participant sorted by intra-individual glycemic variability. [file 1758-5996-5-33-S1.pdf]
